# Obituary

**Published:** 1904-02-15

**Authors:** 


### OBITUARY.

### DR. T. B. WELCH.

Dr. Thomas Bromwell Welch died at his home in Overbrook, Pa., December 29th, 1903, at the age of 78 years. He was born in England, but came to this country when quite young, and was educated in the public schools of Watertown, N. Y. He studied for the ministry and preached for several years and then studied medicine which he practiced for a few years. In 1856 he began practicing dentistry at Winona, Minnesota. In 1865 he located in Vineland, N. J., where he practiced until 1880. In 1881 he went into the dental supply business in Philadelphia. He was the first editor and publisher of the *Items of Interest* which was printed in newspaper form as well as style at first. In his paper he tried to propagate a reform in spelling, which never made much progress and detracted much from the scientific value of his paper. With the dissolution of the Wilmington Dental Manufacturing Co., the *Items of Interest* passed to the Consolidated Manufacturing Co., in New York, and has come to be one of the best dental periodicals published. Dr. Welch has had a varied career, and was widely known because of his public connection with dentistry. but never took part to any considerable extent in the society work of the profession.

